# Physical Activity and Social Relationships on Social Engagement Among Community-Dwelling Older Adults

**DOI:** 10.1093/geroni/igab046.099

**Published:** 2021-12-17

**Authors:** Su-I Hou

**Affiliations:** School of Global Health Management & Informatics, University of Central Florida, University of Central Florida, Florida, United States

## Abstract

This study examined physical activity (FITNESS) and social relationships (FRIENDS) on social engagement among community older adults. Members from two Florida aging-in-village programs participated. Three five-Likert scales were used: A 5-item FITNESS (weight, endurance, strength, flexibility, health), 4-item FRIEND (family, friends, neighbors, communication), and a 3-item social engagement scales (social-leisure activities, stay involved, healthy independent) (Cronbach alphas: .82~.92). Among the 96 participants, 79% were females, 91% were whites, 56% were married, 86% had college education, and 46% living alone. Mean age was 70.7 (SD=10.10). Participants reported at least 30-min. physical activity about 4.2 days per week. Overall social engagement was high (mean=4.38), FITNESS was median (mean=3.46), and FRINED was high (mean=4.19). FITNESS was significant to more 30-min. physical activity. Yet, higher FITNESS, FRIENDS, age, and volunteers were all significant to social engagement. Results has implications on promoting social engagement among older adults participating in aging-in-community programs.

